# Preparation of boron nitride nanosheet-coated carbon fibres and their enhanced antioxidant and microwave-absorbing properties†

**DOI:** 10.1039/c8ra02017e

**Published:** 2018-05-16

**Authors:** Zhichao Xu, Yongjun Chen, Wei Li, Jianbao Li, Hui Yu, Longyang Liu, Gaolong Wu, Tao Yang, Lijie Luo

**Affiliations:** College of Materials and Chemical Engineering, State Key Laboratory of Marine Resource Utilization in South China Sea, Hainan University Haikou 570228 China luolijie4567@163.com

## Abstract

In this study, annealing carbon fibres with boron and FeCl_3_·6H_2_O at elevated temperatures was demonstrated as a novel route to coat carbon fibres with boron nitride (BN) nanosheets. The effect of annealing temperature on the thickness and microstructure of BN coating was investigated. Results showed that BN coating hardly formed at 1000 °C, and uniform BN coating was achieved at 1100 °C and 1200 °C. However, further increasing the temperature to 1250 °C triggered the formation of discretely distributed BN particles on the surface of the BN coating in addition to the formation of a uniform BN coating. The BN coating and particles were constructed by numerous BN nanosheets with a bending and crumpling morphology. The thickness of the BN coating increased with increasing annealing temperature. The oxidation resistance of the carbon fibres dramatically enhanced after BN nanosheets were coated onto the carbon fibre surface. Moreover, given the low dielectric loss tangent of BN, the BN coating can improve the impedance matching of carbon fibres and enhance the microwave-absorbing property of carbon fibres significantly.

## Abstract 2

The coating of BN nanosheets on carbon fibers could enhance the anti-oxidation and microwave absorbing properties of carbon fibers significantly.

## Graphical abstract

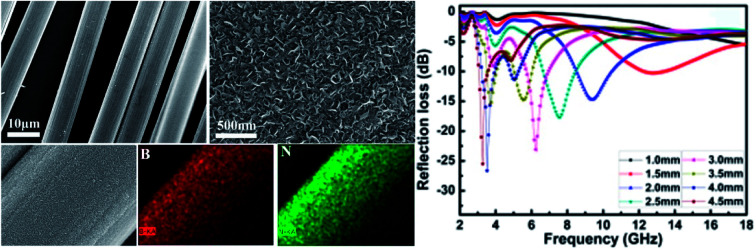

## 1. Introduction

Carbon materials are applied widely in the military, electronic, and infrastructure industries because of their low density, high specific modulus, high specific strength, excellent electrical conductivity, and chemical stability.^1–3^ As a form of carbon, carbon fibres have been used as wave absorbers and reinforced fillers in structural microwave-absorbing materials.^4^ However, carbon fibres are a strong radar reflector against electromagnetic waves in the high-frequency range because of their low electrical resistivity (<10^−3^ Ω m).^4,5^ Carbon fibres are also greatly susceptible to oxidation and can be oxidized at around 400 °C.^6^ Thus, much effort has been exerted to modify carbon fibres with low-cost and light-weight coatings to improve their antioxidant property and microwave absorption property.^5,7,8^ By contrast, most studies mainly focused on the metal coating or metal oxide coating,^9,10^ which cannot effectively dissipate the electromagnetic energy when the application temperature exceeds the coatings' Curie temperatures.^11^

Boron nitride (BN) is a surface-modified material popular for its unique properties, such as low density, high electrical resistivity, good antioxidant property, low dielectric constant, and excellent chemical inertness.^12–14^ BN is also an isostructural analog to carbon with highly similar lattice parameters. These attributes render BN highly suitable in coating carbon fibres and enhancing the antioxidant and microwave-absorbing properties of such material.^8,12,15–17^ Currently, chemical vapor deposition (CVD) and the dip-coating method are mainly used to coat carbon fibres with BN.^14,18–20^ However, the CVD route usually requires hazardous and expensive precursor chemicals, whereas the dip-coating method is a complex process with a long preparation cycle. Therefore, a simple but effective route to coat carbon fibres with BN must be explored. In this study, given the solid-state reaction method developed in our group for the large-scale synthesis of BN micro-nanostructures,^21^ BN nanosheets were coated onto carbon fibres by simple annealing with amorphous boron powder and FeCl_3_·6H_2_O under an NH_3_ atmosphere at elevated temperatures. The thickness and microstructure of the BN coating can be controlled by varying the annealing temperature. The antioxidant and microwave absorption properties of the BN-nanosheet-coated carbon fibres were investigated in detail.

## 2. Experimental

### 2.1 Treatment of carbon fibres

PAN-based carbon fibres (T300, 3K, Toho Tenax, Inc.) were used in this study. A bundle of carbon fibres usually consists of ∼2000 filaments with a typical filament diameter of about 5 μm. Prior to coating, the carbon fibres were heated at 800 °C in a nitrogen atmosphere to remove organic impurities. Then, the fibres were ultrasonically cleaned in acetone for 60 min, followed by drying at 110 °C for 1 h. Herein, clean carbon fibres without sizing were obtained.

### 2.2 The treatment of raw materials

Amorphous boron powders (98% purity, Dandong Chemical Co., Ltd., China) and FeCl_3_·6H_2_O (analytical grade, Aladdin, Shanghai, China) were purchased and used without further purification. The molar ratio was B : FeCl_3_·6H_2_O = 1 : 0.05. First, FeCl_3_·6H_2_O was dissolved in absolute ethyl alcohol, and then B powders were added into the solution. The mixture was stirred in a water bath at 40 °C for 2 h to evaporate the solvent. Afterward, the obtained paste-like mixture was dried at 55 °C to thoroughly remove the ethanol. Finally, a homogeneous mixture containing B and Fe was prepared that provides the boron source for the product.

### 2.3 Preparation of BN coating on carbon fibres

**Fig. 1:**
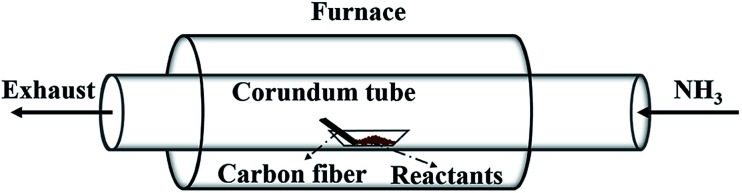
Schematic illustration of the experimental apparatus.

Fig. 1 illustrates the setup for coating BN onto carbon fibres. The carbon fibres used are 2 mm in length and 7 μm in diameter in average. The mixture containing B and Fe prepared above was loaded into an alumina boat placed at the centre of a tube furnace. The clean carbon fibres were placed in the same boat next to the BN precursor along the direction of gas flow. Prior to heating up, high-purity NH_3_ flow was introduced to flush out the residual air in the chamber. Then, the furnace was heated to 1000–1250 °C at a rate of 10 °C min^−1^ under 50 mL min^−1^ NH_3_ flow and maintained for 1 h. Finally, the furnace was cooled naturally under the protection of N_2_ flow. The effect of reaction time (0.5–1.5 h) on the formation of BN coating was conducted and presented in the ESI.†

### 2.4 Characterization

After the carbon fibres were removed from the furnace, they were characterized by Fourier transform infrared spectroscopy (FTIR; Bruker, TENSOR 27 spectrometer), X-ray photoelectron spectroscopy (XPS; ESCALAB 250Xi, Thermo Scientific) with Al Kα radiation, and scanning electron microscopy (SEM; JEM-1200EX, equipped with EDS system). The antioxidant property of the BN-coated carbon fibres was measured in air with a DTA/TGA instrument (STA 449C, NETZSCH). To test the microwave-absorbing property of the samples, the carbon fibres with and without BN coating were cut into short fragments with length of about 2 mm (the average diameters are still about 7 μm because of the thin thickness of BN coating), mixed with molten paraffin (in a mass ratio of 1 : 4), and then pressed into rings with inner diameter × outer diameter × thickness = 3 mm × 7 mm × 2 mm. The electromagnetic parameters were measured by a coaxial line method in the frequency range of 2–18 GHz using a network analyzer (AV3629). Four samples for each batch were measured to gain the average real part of permittivity (*ε*′) and the imaginary part of permittivity (*ε*′′) values.

## 3. Results and discussion

### 3.1 Influence of annealing temperature

**Fig. 2:**
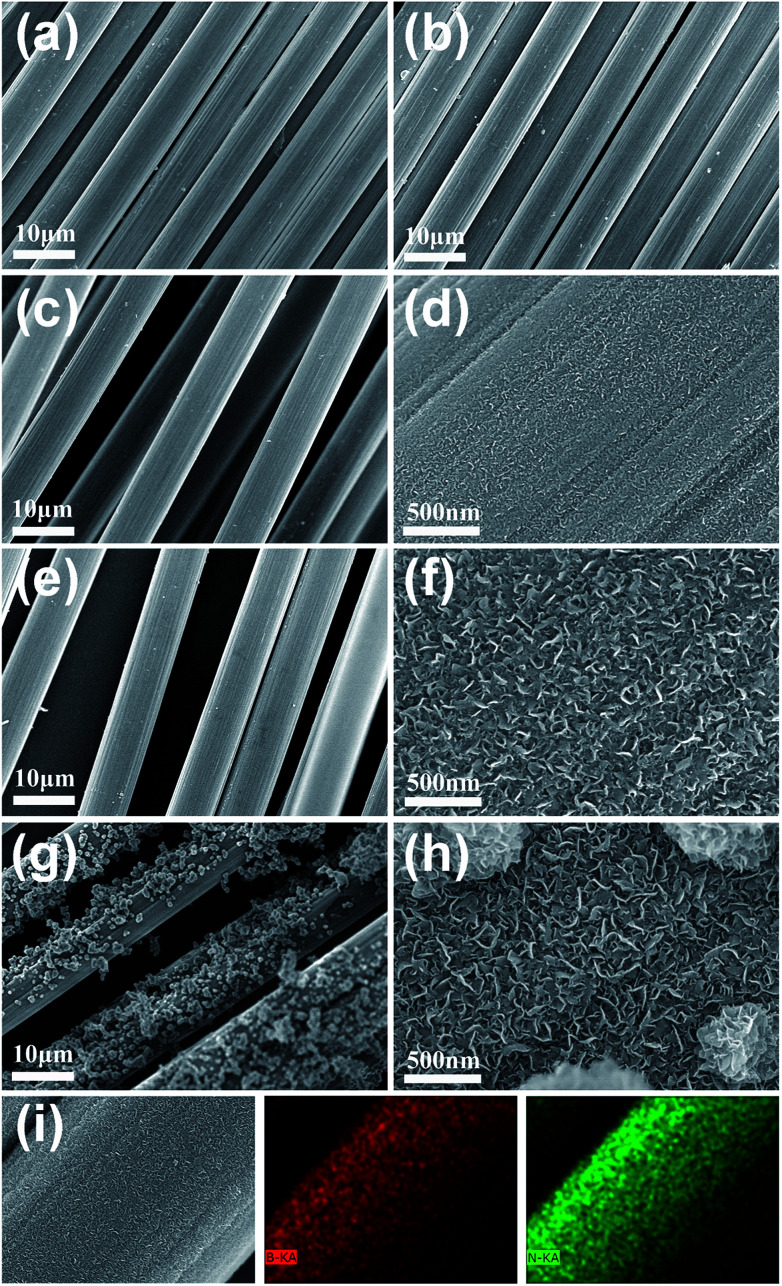
Low and high magnification SEM images of the BN-coated carbon fibres prepared at different temperatures. (a) Without BN coating. (b) 1000 °C. (c) and (d) 1100 °C. (e) and (f) 1200 °C. (g) and (h) 1250 °C. (i) EDS elemental mapping of the BN coating prepared at 1200 °C.

Fig. 2 shows the SEM images of the carbon fibres coated with BN at annealing temperatures of 1000–1250 °C. For comparison, the carbon fibres without BN coating were also characterized by SEM (Fig. 2a), showing the clean surfaces of the carbon fibres. Fig. 2b indicates that no obvious BN coating can be found on the surfaces of the carbon fibres when the annealing temperature was 1000 °C. This result was achieved because very few BN species can be generated through the reaction of B and FeCl_3_ at 1000 °C. With the rise of annealing temperature (*e.g.*, 1100 °C and 1200 °C), an increasing number of BN species were generated and deposited onto the carbon fibres, which resulted in the thickening of BN coatings (Fig. 2c–f). Meanwhile, the magnified images (Fig. 2d and f) show that the coatings were constructed by numerous thin nanosheets. The nanosheets were mostly separated with a bending and crumpling morphology. Moreover, the sizes (thickness and width) of the nanosheets increased with increasing temperature. However, when the annealing temperature was further increased to 1250 °C, many irregular particles formed on the coating surface (Fig. 2g). The magnified image (Fig. 2h) clearly reveals that these particles were also constructed by numerous nanosheets. Fig. 2i shows the elemental mapping of the BN coating prepared at 1200 °C, implying the uniform distribution of B and N elements in the coating.

Thermodynamic calculation shows that the Gibbs free energies of the involved reactions are negative (eqn (1) and (2)), which indicates that the reactions can be occurred thermodynamically in the reaction temperature range 1000–1250 °C. As a result, the thickness of the BN coating increased with the rise of temperature. However, the reactions may have proceeded very violently at 1250 °C and generated an excessive amount of BN species within a short period of time. Hence, surplus BN species deposited directly onto the surfaces of the already formed BN coating and caused the formation of BN nanosheets and even self-assembled BN particles by van der Waals forces. This process is highly similar to that of BN-nanosheet-assembled microwires we reported previously.^21^

1 FeCl_3_ + B → BCl_3_ (g) + Fe, Δ*G* (1000 – 1250 °C) = −140.63 to −152.05 kJ

2 BCl_3_ (g) + NH_3_ (g) → BN + 3HCl (g), Δ*G* (1000 – 1250 °C) = −201.15 to −226.04 kJ

### 3.2 FTIR and XPS analyses

**Fig. 3:**
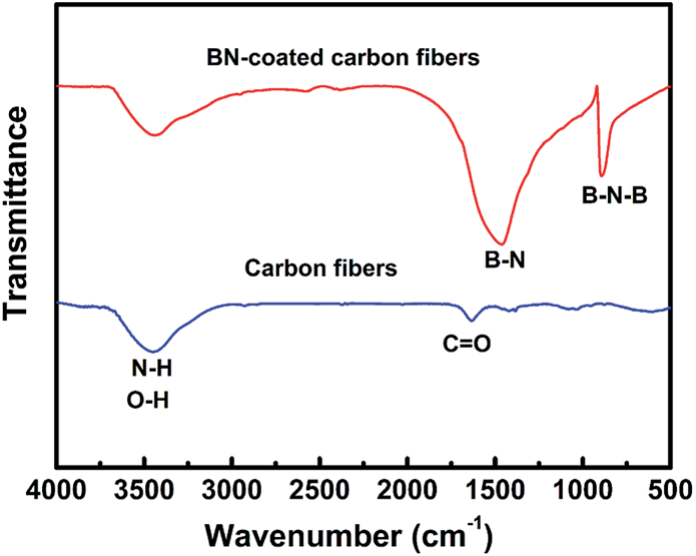
FT-IR spectra of the clean carbon fibres and BN-coated carbon fibres fabricated at 1200 °C.

Fig. 3 shows the FTIR spectra of carbon fibres with and without BN coating. The two absorption bands located at 1384 and 802 cm^−1^ were detected in the BN-coated carbon fibres prepared at 1200 °C. These bands were attributed to the B–N in-plane stretching vibrations and the B–N–B out-of-plane bending vibrations, respectively.^22,23^ Meanwhile, the band at 3407 cm^−1^ can be assigned to the stretching vibration of O–H and N–H bonds because of the absorbed water on the surfaces of BN-coated carbon fibres.^24^

**Fig. 4:**
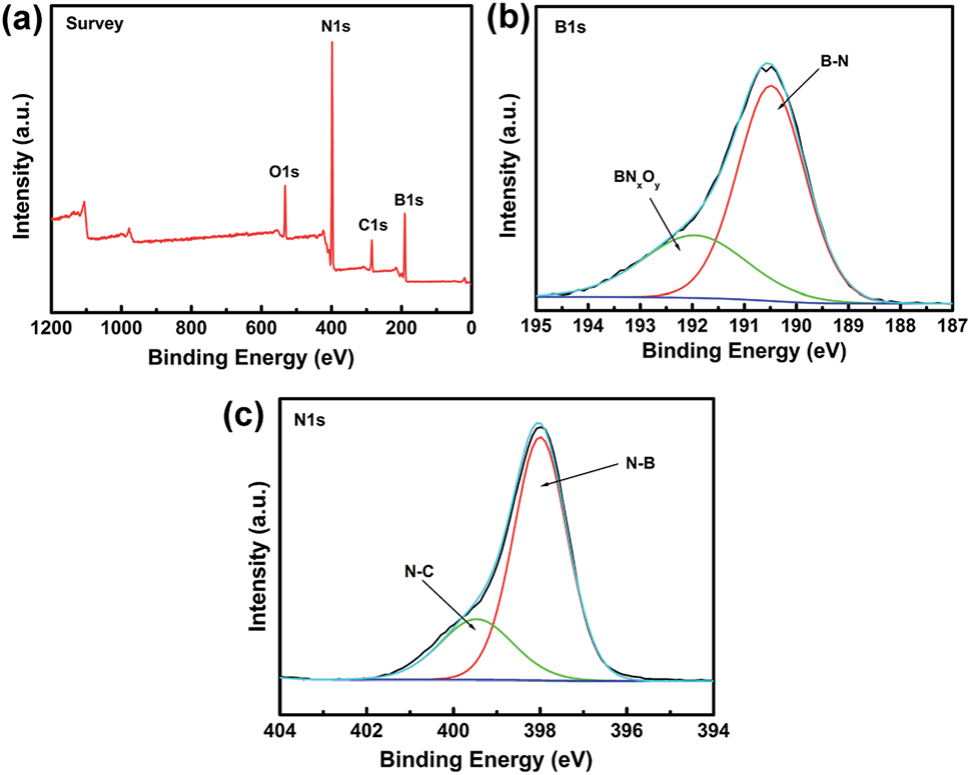
XPS spectra of the BN-coated carbon fibres fabricated at 1200 °C. (a) Survey scan spectrum. (b) Fitted B 1s spectra. (c) Fitted N 1s spectra.

XPS is a powerful spectroscopic technique for characterizing surfaces with chemical bonding. Therefore, XPS was used to obtain additional information on the chemical composition of BN coating fabricated at 1200 °C (Fig. 4). All XPS data were corrected by using the binding energy (BE) of C–C at 284.6 eV.

Fig. 4a shows the XPS survey scan of BN coating and indicates the presence of B, N, O, and C elements in the sample. The C signal can be ascribed to the carbon fibre or adventitious hydrocarbon from the XPS instrument itself.^25^ The narrow XPS spectra of B 1s and N 1s are shown in Fig. 4b and c, respectively. The B 1s peak at 190.4 eV and the N 1s peak at 398.2 eV were identified as B–N bonding and matched those reported for bulk h-BN.^26^ The B 1s band was divided into two fine peaks located at 190.4 and 191.9 eV, respectively. The 190.4 eV peak was assigned to the B–N bonding,^27,28^ whereas the peak at 191.9 eV was assigned to the substitution of N by O in the BN lattice, *i.e.*, the formation of O–B or O–B–N bonds of BN_*x*_O_*y*_.^29^ The N 1s peak can also be divided into two fine peaks centred at 397.9 and 399.4 eV, respectively. The state of N at 397.9 eV can be ascribed to the N–B bonds, whereas the peak at 399.4 eV was attributed to N–C bonds, which agreed with the results reported in literature.^30^

### 3.3 Oxidation resistance of BN-coated carbon fibres

**Fig. 5:**
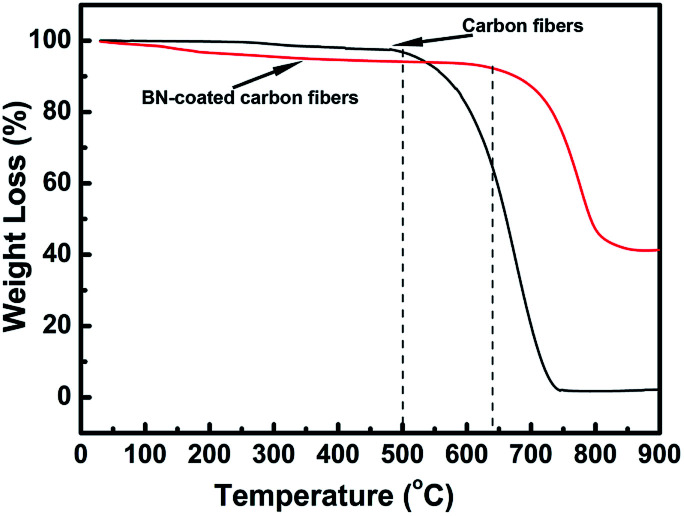
TGA curves of clean carbon fibres and BN-coated carbon fibres.

The oxidation resistance of the carbon fibres with and without BN coating was investigated by performing TGA test in air from room temperature to 900 °C (Fig. 5). Research found that the carbon fibres without BN coating began to be oxidized at about 500 °C and underwent rapid weight loss when the temperature was increased. When the temperature was increased to 740 °C, these carbon fibres were nearly completely oxidized with only about 1% residual weight. However, the starting oxidation temperature and end-oxidation temperature for the BN-coated carbon fibres increased to about 640 °C and 850 °C, respectively. Thus, the antioxidant property of the carbon fibres was improved efficiently by BN coating.

### 3.4 Microwave-absorbing property

**Fig. 6:**
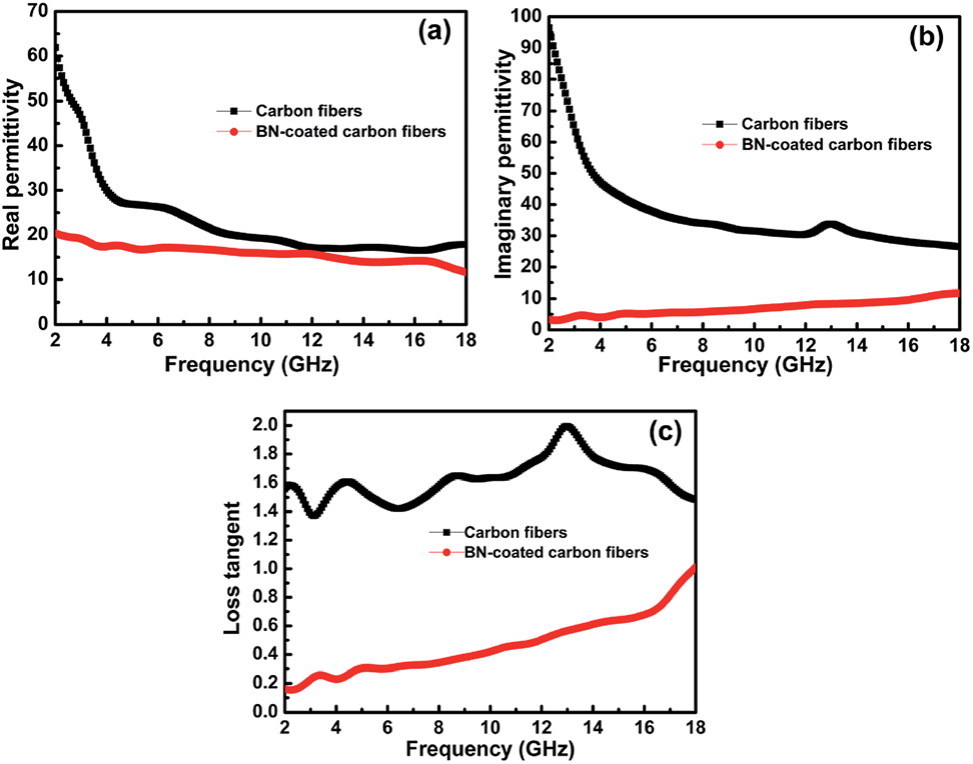
Frequency dependence of (a) the real parts and (b) the imaginary parts of permittivity, and the corresponding dielectric loss (c) of the carbon fibres with and without BN coating.

According to Debye theory, the real part of permittivity (*ε*′) and the imaginary part of permittivity (*ε*′′) represent the storage ability and the dissipation ability of electromagnetic wave energy, respectively. Fig. 6a and b show the *ε*′ and *ε*′′ of the clean carbon fibres and BN-coated carbon fibres measured at room temperature in the frequency range of 2–18 GHz. The *ε*′ and *ε*′′ values of the carbon fibres decreased obviously after BN coating, especially when the frequency was less than 8 GHz. This decrease may be attributed to the separation of dielectric relaxation and space charge polarization of carbon fibres after BN coating because BN possesses a high electrical resistivity.^31^ In addition, the extremely low dielectric constant and low dielectric loss of BN can also help decrease the complex permittivity of carbon fibres.^1^

Herein, the microwave attenuation of the samples was mainly attributed to dielectric loss rather than magnetic loss.^32^Fig. 6c shows that the dielectric loss tangent (tan *δ*) of the BN-coated carbon fibres was smaller than that of the clean carbon fibres in the measured frequency range. To obtain good impedance matching, the values of tan *δ* should be approximately equal to the magnetic tangent loss.^33^ Carbon fibres reflect microwaves highly because their dielectric loss tangent is obviously higher than their magnetic loss tangent. Hence, the BN coating with a low dielectric loss tangent can narrow the gap between the dielectric and magnetic loss tangents of carbon fibres.

**Fig. 7:**
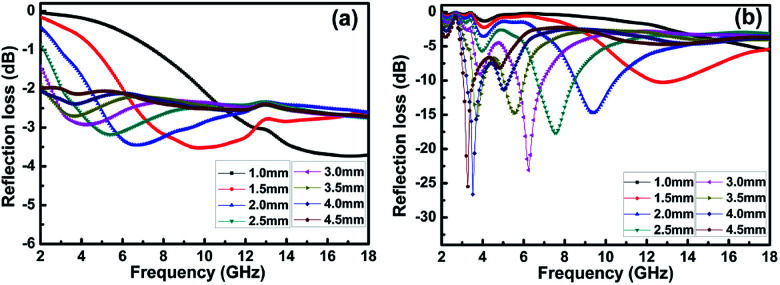
Frequency dependence of RL for (a) clean carbon fibres and (b) BN-coated carbon fibres with different thickness.

To further describe the microwave-absorbing property of the samples, the reflection loss (RL) of the carbon fibres with and without BN coating was calculated in accordance with the following equations by using the single-layer model:^33,34^3
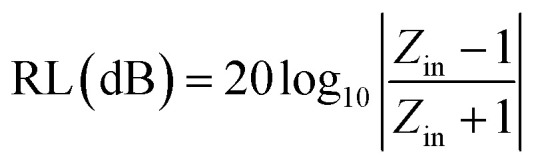
4
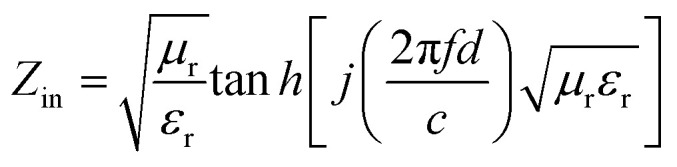
where *Z*_in_ is the normalized input impedance relative to the impedance in free space, *f* is the microwave frequency, *c* is the light velocity, *d* is the thickness of the absorber, and *ε*_r_ and *μ*_r_ are the complex permittivity and complex permeability of materials, respectively. Therefore, the *R* values *versus* frequency can be evaluated at a specified thickness. Fig. 7 shows the calculated RL of the samples (thickness 1–4.5 mm) with and without BN coating in the frequency range of 2–18 GHz. As shown in Fig. 7a, the RL values of the clean carbon fibres were larger than −4 dB in the frequency range of 2–18 GHz. This poor microwave absorption performance was mainly caused by the poor impedance matching and most microwaves were reflected by the front of the sample surface.^35^Fig. 7b illustrates that the BN coating improves the microwave-absorbing property of the carbon fibres significantly, and the RL values are listed in Table 1. Meanwhile, the dependence of RL values on sample thickness can be concluded.

**Table 1:** The electromagnetic microwave absorption properties of BN/carbon fibre based absorbers with different thickness

Thickness (mm)	Min RL value (dB)	Frequency (GHz)
1.0	−5.60	18.00
1.5	−10.40	12.73
2.0	−14.85	9.32
2.5	−17.72	7.56
3.0	−22.97	6.17
3.5	−15.57	3.68
4.0	−26.79	3.45
4.5	−25.52	3.24

The lowest RL can reach −26.8 dB at 3.5 GHz with a sample thickness of 4.0 mm. The widest frequency range where *R* is lower than −10 dB was 8.6–10.4 GHz when the sample thickness was 2.0 mm. In addition, RL values less than −10 dB can be gained in the 2.9–13.4 GHz range with sample thicknesses of 1.5–4.5 mm. This result means that the microwave absorption performance of the carbon fibres in different frequency bands can be adjusted by varying the sample thickness. A material with an RL value less than −20 dB is an excellent absorber because this value corresponds to a microwave absorption of 99.99%.^34^ Therefore, we conclude that the microwave-absorbing property of carbon fibres can be significantly enhanced by BN coating through the reduction of microwave reflection.

## 4. Conclusions

BN coatings have been successfully deposited on the surface of carbon fibres by annealing amorphous boron powder with FeCl_3_·6H_2_O under flowing ammonia atmosphere at 1100–1250 °C. The thickness of coating increases with the rise of annealing temperature and uniform coatings can be obtained at 1100–1200 °C. The coatings consist of numerous BN nanosheet which are mostly separated with a bending and crumpling morphology. Irregular BN particles are formed and distributed discretely on the BN coating when the temperature increases to 1250 °C, which are also constructed of BN nanosheets. The antioxidation property of carbon fibres is improved significantly by BN coating, with start oxidization temperatures increasing from 500 to 640 °C. In addition, the complex permittivity of BN-coated carbon fibres decreases greatly due to the extremely low dielectric constant, low dielectric loss and high electrical resistivity of BN coatings. The carbon fibres with BN coating show a strong absorption peak at 3.5 GHz, where the lowest reflectivity can reach −26.8 dB. Moreover, the reflection loss less than −10 dB is over the range of 2.9–13.4 GHz, indicating an excellent microwave absorbing property of the BN-coated carbon fibres. Therefore, it is an effective modification approach to enhance the oxidation resistance and microwave absorbing properties of carbon fibres.

## Conflicts of interest

There are no conflicts to declare.

## Supplementary Material

RA-008-C8RA02017E-s001
