# Supplementary material for: Preparation of boron nitride nanosheet-coated carbon fibres and their enhanced antioxidant and microwave-absorbing properties
Source: RSC Adv. 2018 May 16;8(32):17944–9. doi: 10.1039/c8ra02017e (PMC9080507; doi:10.1039/c8ra02017e)
Supplement: RA-008-C8RA02017E-s001 [file RA-008-C8RA02017E-s001.pdf]

Electronic Supplementary Material (ESI) for RSC Advances.
This journal is © The Royal Society of Chemistry 2018

Supporting Information

Preparation of boron nitride nanosheet-coated carbon fibers and their enhanced antioxidant and microwave-absorbing properties

Zhichao Xu, [a] Yongjun Chen,[a] Wei Li, [a] Jianbao Li, [a] Hui Yu, [a] Longyang Liu, [a] Gaolong Wu, [a] Tao Yang [a] and Lijie Luo*[a]

College of Materials and Chemical Engineering, State Key Laboratory of Marine Resource Utilization in South China Sea, Hainan University, Haikou 570228, China

*The corresponding author. Email: luolijie4567@163.com (L.J. Luo)

In order to determine the repeatability of the experiments, we additionally conducted the experiments respecting the influence of reaction time on the formation of BN coating.

Fig. S1 shows the BN coating prepared with different reaction times. It can be seen that the thickness of BN coating increases with the increase of reaction time, as well the size (including thickness and length) of the BN nanosheets. However, if the reaction time is longer enough (e.g. 1.5 h), the BN nanosheets will be self-assembled into microparticles which are constructed by numerous nanosheets. This phenomenon is very similar to the results caused by the increase of reaction temperature which are discussed in the main text.

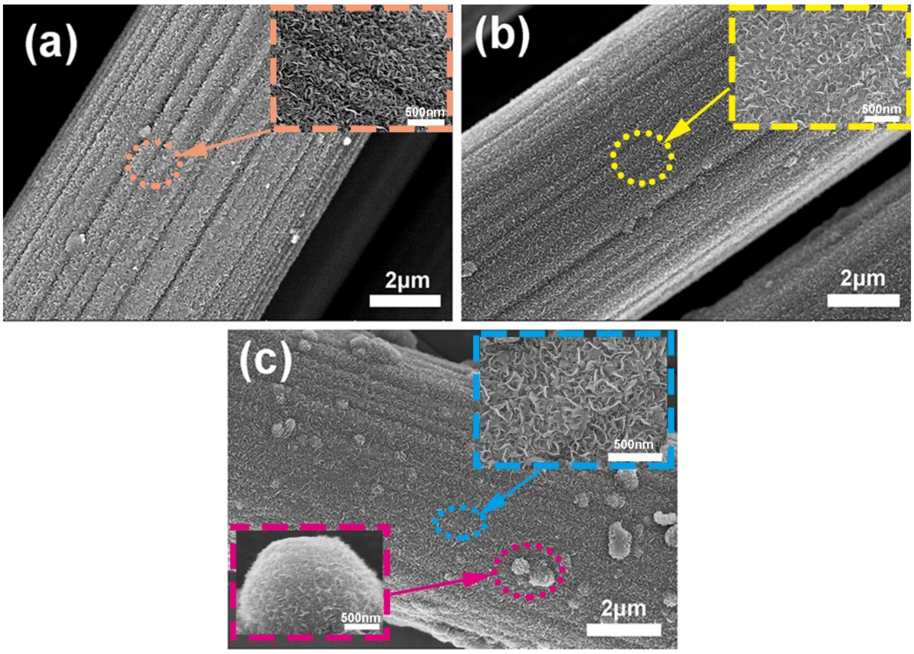

Fig. S1 SEM images of BN coating prepared with different reaction times. (a) 0.5 h. (b) 1 h. (c) 1.5 h.
